# First Report on Mitochondrial Gene Rearrangement in Non-Biting Midges, Revealing a Synapomorphy in *Stenochironomus* Kieffer (Diptera: Chironomidae)

**DOI:** 10.3390/insects13020115

**Published:** 2022-01-21

**Authors:** Chen-Guang Zheng, Zheng Liu, Yan-Min Zhao, Yang Wang, Wen-Jun Bu, Xin-Hua Wang, Xiao-Long Lin

**Affiliations:** 1 College of Life Sciences, Nankai University, Tianjin 300071, China; chenguangzheng@nankai.edu.cn (C.-G.Z.); wenjunbu@nankai.edu.cn (W.-J.B.); xhwang@nankai.edu.cn (X.-H.W.); 2 Geological Museum of China, Beijing 100083, China; armylan@163.com; 3 State Key Laboratory of Environmental Criteria and Risk Assessment, Chinese Research Academy of Environmental Sciences, Beijing 100012, China; zhaoym@craes.org.cn; 4 Department of Plant Protection, College of Horticulture and Landscape, Tianjin Agricultural University, Tianjin 300392, China; wy18822300279@163.com

**Keywords:** mitochondrial genome, gene rearrangement, chironomid, phylogeny

## Abstract

**Simple Summary:**

Gene rearrangement is an additional type of data to support relationships of taxa, with rearrangement synapomorphies identified across multiple orders and at many different taxonomic levels. The concept to use mitochondrial gene rearrangements as phylogenetic markers has been proposed since the mid-1980s, the synapomorphic gene rearrangements have been identified from many lineages. However, mitochondrial gene rearrangement has never been observed in the non-biting midges (Diptera: Chironomidae). Here, seven new mitogenomes of the genus *Stenochironomus* were sequenced and analyzed. Coupled with published data, phylogenetic analyses were performed within Chironominae. The present study showed that mitogenomes of *Stenochironomus* are showing a higher A+T bias than other chironomid species. A synapomorphic gene rearrangement that the gene order rearranges from *trnI-trnQ-trnM* to *trnI-trnM-trnQ* was identified within *Stenochironomus*, which is the first instance of mitochondrial gene rearrangement discovered in the Chironomidae. The monophyly of the genus *Stenochironomus* was strongly supported by mitogenomes. Our study provides new insights into the mitochondrial gene order of Chironomidae, and provides a valuable resource for understanding synapomorphic gene rearrangements.

**Abstract:**

(1) Background: Gene rearrangement of mitochondrial genome, especially those with phylogenetic signals, has long fascinated evolutionary biologists. The synapomorphic gene rearrangements have been identified across multiple orders and at many different taxonomic levels, supporting the monophyletic or systematic relationships of related lineages. However, mitochondrial gene rearrangement has never been observed in the non-biting midges (Diptera: Chironomidae); (2) methods: in this study, the complete mitogenomes of seven *Stenochironomus* species were sequenced and analyzed for the first time; (3) results: each mitogenome of *Stenochironomus* contains 37 typical genes and a control region. The whole mitogenomes of *Stenochironomus* species exhibit a higher A+T bias than other published chironomid species. The gene order rearranges from *trnI-trnQ-trnM* to *trnI-trnM-trnQ* in all the seven mitogenomes of *Stenochironomus*, which might be act as a synapomorphy of the genus, supporting the monophyletic of *Stenochironomus* species. In addition, another derived gene cluster: *trnA-trnG-ND3-trnR* exists in *Stenochironomus tobaduodecimus.* The derived gene orders described above are the first case of mitochondrial gene rearrangement in Chironomidae. Coupled with published data, phylogenetic relationships were reconstructed within Chironominae, and strongly supported the monophyly of *Stenochironomus*; (4) conclusions: our study provides new insights into the mitochondrial gene order of Chironomidae, and provides a valuable resource for understanding the synapomorphic gene rearrangements.

## 1. Introduction

**Figure 1:**
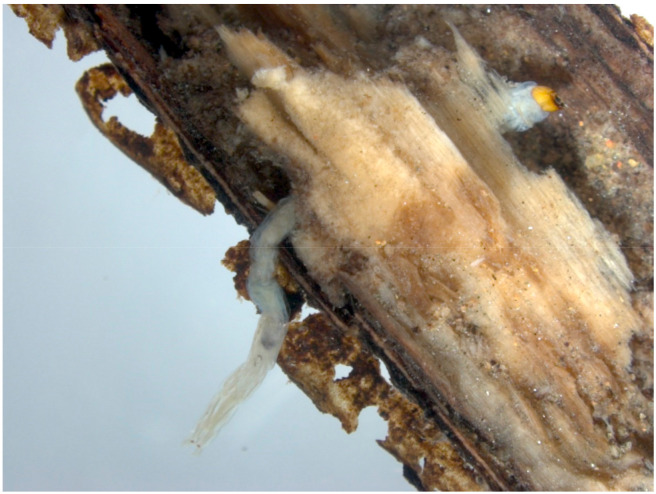
Larva of *Stenochironomus okialbus* in immersed wood from Zhejiang, China.

Gene rearrangement of mitochondrial genome (mitogenome) has long fascinated evolutionary biologists, and insects are one of the most studied organisms [1,2,3]. The typical mitogenome of insect is a 14–20 kb circular molecule, containing 37 genes (13 protein-coding genes, two ribosomal RNA genes, and 22 transfer RNA genes) and a control region on a single chromosome [4] characterized by the small genome size, maternal inheritance, low sequence recombination, and fast evolution rates [1,5,6]. Benefited by the high-throughput sequencing technology, nucleotide sequences of mitogenomes are now widely used in phylogenetic and evolutionary studies [7,8,9]. Besides, gene rearrangement is an additional type of data to support relationships of taxa, with rearrangement synapomorphies identified across multiple orders and at many different taxonomic levels [1,10,11]. Since the concept of using mitochondrial gene rearrangements as phylogenetic markers has been proposed in the mid-1980s [3], the synapomorphic gene rearrangements have been identified in many taxa, supporting the monophyletic or systematic relationships of related lineages [9,12,13]. In insect mitogenomes, patterns of gene arrangement are usually conserved within lineages [6], but gene rearrangements have also been observed involving tRNA and PCG within many orders, such as Blattodea [14], Ephemeroptera [15,16], Hemiptera [17,18], Hymenoptera [12,19], Lepidoptera [20], Mantodea [21,22], Orthoptera [23,24], Phthiraptera [25], Psocoptera [9], and Thysanoptera [26]. For the mitogenomes of Diptera, gene rearrangements have been detected within several families, e.g., Calliphoridae [27], Cecidomyiidae [28], and Mycetophilidae [29]. Published mitogenome data of Chironomid are relatively rare compared with other insects of Diptera. Previous studies have focused on the genome organization and the application of nucleotide sequence information in phylogenetic analysis [30,31,32,33,34,35,36,37]. However, mitochondrial gene rearrangement has never been reported in the Dipteran family Chironomidae. In the current study, we found mitochondrial gene rearrangements in the genus *Stenochironomus* Kieffer for the first time in Chironomidae. *Stenochironomus* is species-diverse, and occurs in all zoogeographic regions except Antarctica, with more than 100 named species [38]. The larvae of *Stenochironomus* (Figure 1) are found mining leaves or immersed wood in standing and flowing waters [38]. Due to its strictly mining habit which contributes to decomposition of wood, leaves, and aquatic macrophytes, *Stenochironomus* is regarded as important bioindicator in freshwater ecosystems [39,40,41].

Prior to this study, the mitogenomic characteristics of *Stenochironomus* have never been studied. We sequenced and annotated the complete mitogenomes of seven species of *Stenochironomus*. The mitogenomic organization, evolutionary rates, and gene rearrangement pattern within *Stenochironomus* were revealed. Coupled with published data, phylogenetic relationships of Chironominae were reconstructed based on mitogenomes to explore the monophyly of *Stenochironomus*.

## 2. Materials and Methods

### 2.1. Taxon Sampling and DNA Extraction

Seven species of *Stenochironomus* were used for mitogenome sequencing (Table 1). Specimens were preserved in ethanol (85% for adults, 95% for immature) and stored in a freezer at −20 °C in the College of Life Sciences at Nankai University (Tianjin, China). The total genomic DNA was the extracted thorax of adult and larva using a Qiagen DNA Blood and Tissue Kit (Qiagen, Hilden, Germany) following the manufacturer’s protocol. The DNA and vouchers of the species are deposited at the College of Life Sciences, Nankai University, Tianjin, China.

### 2.2. Sequencing and Mitogenome Assembly

Whole mitogenomes were sequenced individually using the Illumina NovaSeq 6000 platform with a 350-bp insert size and a paired-end 150-bp sequencing strategy at Novogene Co., Ltd. (Beijing, China). Adapter sequences and low-quality reads were trimmed with Trimmomatic 0.36 [43], and finally about two Gb of clean data were obtained for each sample. A de novo assembly was performed using IDBA-UD [44] with minimum and maximum k values of 40 and 120 bp, respectively. The partial COI sequence for each species was downloaded from GenBank, and served as the “bait” reference to acquire the targeted mitogenome sequence from the pooled sequencing file. To check the accuracy of the assembly, clean reads were mapped onto the obtained mitogenome sequences using Geneious 2020.2.1 [45].

### 2.3. Genome Annotation and Sequence Analyses

The MITOS2 webserver (available at http://mitos2.bioinf.uni-leipzig.de/index.py, access on 24 May 2021) was used to identify transfer RNA (tRNA) genes based on the invertebrate mitochondrial genetic code. Protein coding genes (PCGs) and ribosomal RNA (rRNA) genes were annotated by aligning with homologous regions of other published chironomid mitogenomes using Geneious 2020.2.1. Newly sequenced mitogenomes were submitted to GenBank (accession numbers: OL742440, OL742441, OL753645–753649). The CG View server V 1.0 [46] was used to draw the mitogenome maps. Nucleotide compositions of the whole mitogenome and individual genes were calculated by MEGA X [47]. The bias of the nucleotide composition was measured by AT-skew [(A-T)/(A+T)] and GC-skew [(G-C)/(G+C)]. The codon usage of PCGs were computed by MEGA. The non-synonymous substitution rate (Ka) and synonymous substitution rate (Ks) of PCGs were calculated with DnaSP 6.12.03 [48].

### 2.4. Phylogenetic Analyses

Phylogenetic analyses were conducted using the seven newly sequenced *Stenochironomus* mitogenomes and five Chironominae species available in GenBank as ingroup taxa (Table 1). One Orthocladiinae species (*Rheocricotopus villiculus*) was chosen as an outgroup. Individual genes of PCGs were aligned using muscle implemented in MEGA based on amino acid sequences, and then concatenated using SequenceMatrix v1.7.8 [49]. A total of three datasets were prepared for phylogenetic analyses: PCG123 (all three codon positions of the 13 PCGs), PCG12 (the 1st and 2nd codon positions of the 13 PCGs), and AA (amino acid sequences of the 13 PCGs). PartitionFinder 2.0 [50] was used to select the best partitioning scheme and best-fit substitution model. Bayesian inference (BI) and maximum likelihood (ML) methods were used for phylogenetic analyses. BI analysis was conducted using MrBayes 3.2.7a [51] with substitution model in Supplementary Table S1. Two simultaneous Markov chain Monte Carlo (MCMC) runs of 10,000,000 generations were conducted, trees were sampled every 1000 generations and the first 25% of trees discarded as burn-in. The convergence of runs was checked using Tracer 1.7 [52]. The ML analysis was performed using IQ-TREE 1.6.10 [53] with the best-fit substitution model and 1000 bootstrap replicates.

## 3. Results and Discussion

### 3.1. General Features of Stenochironomus Mitogenomes

**Figure 2:**
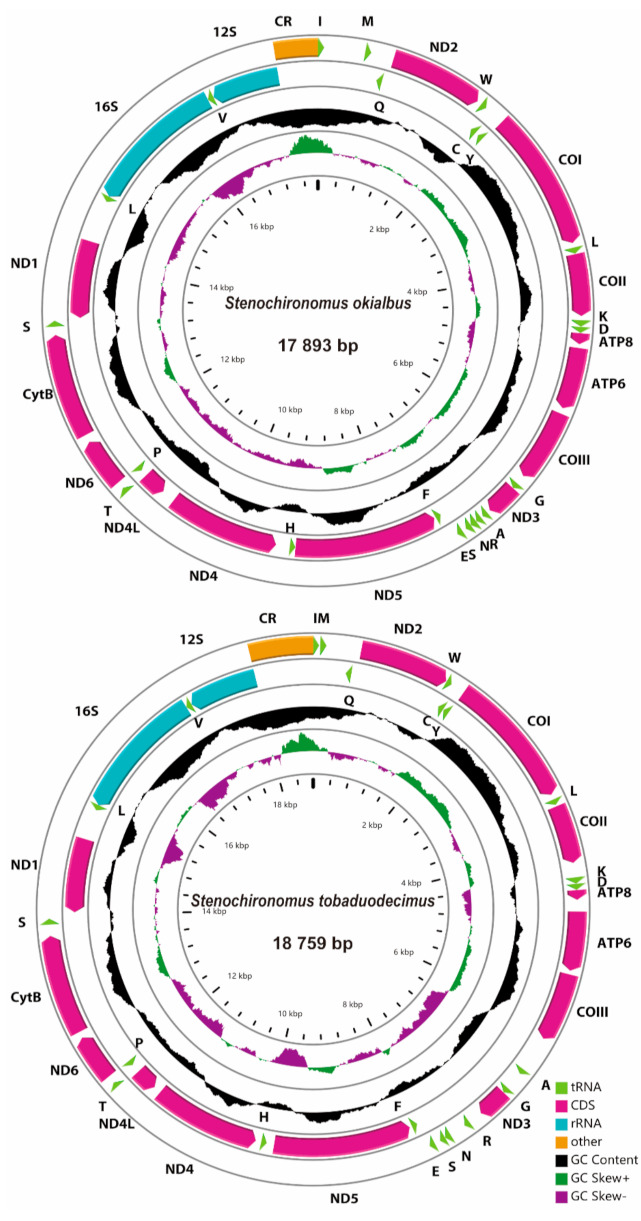
Mitogenome maps of the represented species of *Stenochironomus*. The names of PCGs and rRNAs are indicated by standard abbreviations, while names of tRNAs are represented by a single letter abbreviation. The first circle shows the gene arrangement and arrows indicate the orientation of gene transcription. Red, blue, green, and yellow arrows refer to PCGs, rRNAs, tRNAs, and the control region, respectively. The second circle indicates the GC content, which is plotted as the deviation from the average GC content of the entire sequence. The third circle shows the GC-skew, which is plotted as the deviation from the average GC-skew of the entire sequence. The innermost circle shows the sequence length.

Seven mitogenomes of *Stenochironomus* were newly sequenced with the length range from 17,694 bp in *Stenochironomus* sp. 2CZ to 18,759 bp in *Stenochironomus tobaduodecimus* (Figure 2). Among them, the control regions of *Stenochironomus gibbus* and *Stenochironomus* sp. 2CZ failed to complete sequencing due to the complicated structure and high AT content. The entire length of mitogenomes of the *Stenochironomus* species is larger than other published chironomid species, mainly due to the large number of intergenic spacers [30,31,32,33]. Each mitogenome of *Stenochironomus* contains 37 typical genes (13 PCGs, two rRNAs, and 22 tRNAs) and one control region. Among these genes, four PCGs, eight tRNAs, and two rRNAs are coded on the minority strand (N strand), while the other genes are coded on the majority strand (J strand) (Figure 2).

The whole mitogenomes of *Stenochironomus* are significantly biased toward A and T with the A+T content range from 81.7% in *Stenochironomus tobaduodecimus* and *Stenochironomus* sp. 1CZ to 83.6% in *Stenochironomus* sp. 3CZ (Table 2), showing a stronger A+T bias than other chironomid species [30,31,32,33]. Among the mitogenomes of *Stenochironomus*, the 3rd codon of PCGs and the control region exhibit the highest A+T content while the 1st and 2nd codons of PCGs have the lowest A+T content (Table 2). The whole mitogenomes of all the seven *Stenochironomus* species exhibit positive AT-skew (0.01 to 0.02) and negative GC-skew (−0.34 to −0.21), except for *Stenochironomus tobaduodecimus* and *Stenochironomus zhengi* (Table 2).

**Figure 3:**
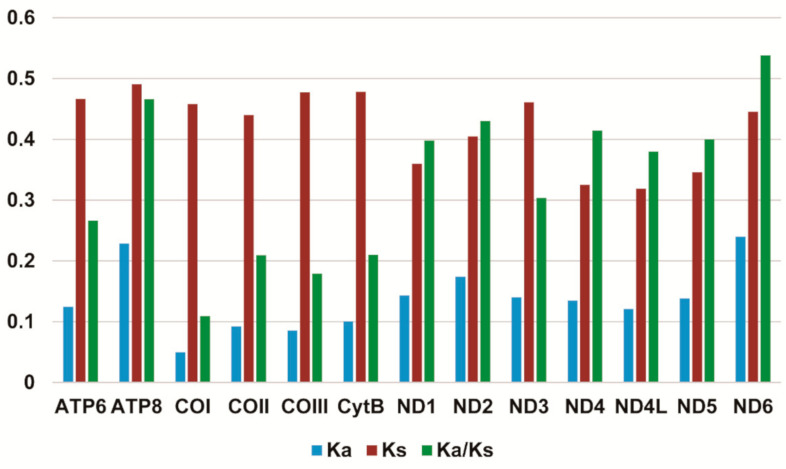
Evolution rate of each PCG of the seven *Stenochironomus* mitogenomes. Ka refers to non-synonymous substitution rate, Ks refers to synonymous substitution rate, Ka/Ks refers to evolution rate of each PCG.

Most PCGs in *Stenochironomus* mitogenomes initiate with a standard start codon ATN (N represents one of four nucleotides, A, T, C, G). While the start codon of COI is TTG in *Stenochironomus zhengi* and *Stenochironomus* sp. 1CZ. The start codon of ND5 in *Stenochironomus zhengi*, *Stenochironomus* sp. 1CZ, and *Stenochironomus* sp. 3CZ is GTG (Supplementary Table S2). All PCGs in *Stenochironomus* mitogenomes end with TAA or TAG as the termination codon (Supplementary Table S2). The total codon numbers, except the termination codons among *Stenochironomus* mitogenomes range from 3707 in *Stenochironomus* sp. 1CZ to 3716 in *Stenochironomus* sp. 3CZ (Supplementary Table S3). The highest and lowest frequent codon families are Phe and Cys, respectively (Supplementary Figure S1), which is congruent with those of previously published chironomid species [32,33,34,35]. The Ka/Ks value (ω) is used to test for signatures of natural selection. The ω value of all PCGs in *Stenochironomus* mitogenomes is less than 1 (Figure 3), suggesting that they are under purifying selection. ND6 exhibits the highest ω value among the 13 PCGs of *Stenochironomus* mitogenomes (Figure 3), while ATP8 evolves at the fastest rate in previously published chironomid species [32,33].

Each mitogenome of *Stenochironomus* contains 22 typical tRNA genes, with A+T content ranging from 83.4% to 85.1% (Table 2). The nucleotide skew of tRNA genes among *Stenochironomus* mitogenomes is consistent, the concatenated tRNA genes exhibit a positive AT-skew, and negative GC-skew (Table 2). The A+T content of 12S rRNA and 16S rRNA genes range from 86.1% to 89.4%, and 87.2% to 89.4%, respectively. The 12S rRNA exhibits a negative AT-skew and negative GC-skew, while the 16S rRNA exhibits a positive AT-skew and negative GC-skew in most *Stenochironomus* mitogenomes (Table 2).

### 3.2. Gene Rearrangement

**Figure 4:**
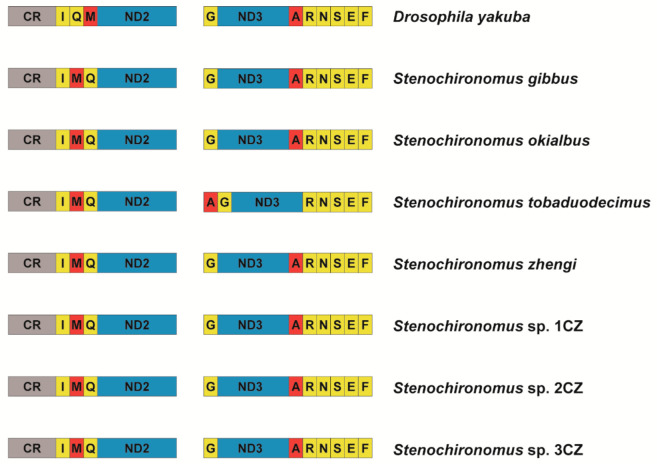
Gene rearrangement of *Stenochironomus* mitogenomes. The names of PCGs are indicated by standard abbreviations and the names of tRNAs are represented by a single letter abbreviation.

The gene arrangement of mitogenomes is conservative in most groups of Diptera [30,54,55,56]. New gene orders have only been reported in a few taxa, for example: The *trnI* gene inverted and transposed from the position between the control region and the *ND2* gene to the block of tRNA genes between *ND3* and *ND5* in two gall midges [28], and the midge *Arachnocampa flava* has an inversion of the *trnE* gene [30]. Prior to this study, no examples of gene rearrangement were reported from mitogenomes of non-biting midge species. The gene order rearranges from *trnI-trnQ-trnM* to *trnI-trnM-trnQ* in all the seven *Stenochironomus* mitogenomes and the *trnA gene* moves to upstream, forming a new gene cluster: *trnA-trnG-ND3-trnR* in *Stenochironomus tobaduodecimus* (Figure 4), which is the first instance of mitochondrial gene rearrangement discovered in Chironomidae. Previous studies have shown that gene rearrangement can act as a synapomorphy and be shared within different taxonomic levels [1,11,57,58]. In this study, the gene rearrangement (*trnI-trnM-trnQ*) is discovered in all the seven *Stenochironomus* mitogenomes, which might act as a synapomorphy of the genus, supporting the monophyletic of the *Stenochironomus* species.

In the insect mitogenome, the gene cluster *trnI-trnM-trnQ* is one of the regions with the highest probability of gene rearrangement. Many gene rearrangement events related to this region have been reported, including gene duplication [2,20,59], and gene translocation [21,60]. The derived gene order *trnI-trnM-trnQ,* same as in this study have been observed in 14 species belonging to 5 different orders in previous studies [2]. The gene cluster *trnI-trnM-trnQ* is adjacent to the control region of mitogenome, and transcription of the entire mitogenome starts from this region. The probability of rearrangement may be higher at the beginning of transcription [1,2,11]. The six-tRNA gene cluster *trnA-trnR-trnN-trnS-trnE-trnF* is also a gene rearrangement the hotspot region. Cases of gene rearrangement in this region have been reported in many different orders of insects [1,2].

### 3.3. Phylogenetic Relationships

**Figure 5:**
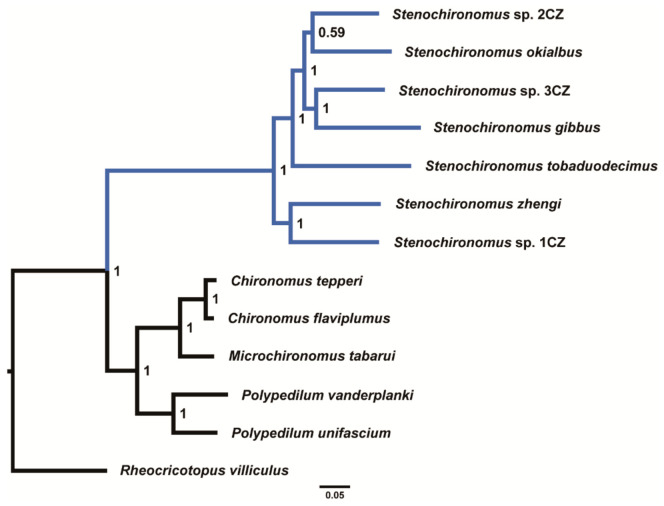
Phylogenetic tree of Chironominae based on dataset AA. Numbers at the nodes are BI.

The phylogenetic analyses were performed based on the concatenated nucleotide sequences of 13 PCGs. All phylogenetic trees based on different datasets show that the seven *Stenochironomus* species form a monophyletic group with strong support (Figure 5 and Supplementary Figures S2–S4). Our study reveals that the mitogenomes of *Stenochironomus* are useful for phylogenetic inference. Although mitogenomes have poor phylogenetic signals at the subfamily level of Chironomidae [33], mitogenomes are still useful for phylogeny at the genus level within Chironomidae according to the present and recent studies [32].

## 4. Conclusions

In this study, seven new mitogenomes of the genus *Stenochironomus* were sequenced and analyzed. Coupled with published data, phylogenetic analyses were performed within Chironominae. The present study showed that mitogenomes of *Stenochironomus* show a higher A and T bias than other chironomid species. A synapomorphic gene rearrangement that the gene order rearranges from *trnI-trnQ-trnM* to *trnI-trnM-trnQ* was identified within *Stenochironomus*, which is the first instance of mitochondrial gene rearrangement discovered in Chironomidae. The monophyly of the genus *Stenochironomus* was strongly supported by mitogenomes.

## Figures and Tables

**Table 1:** Taxonomic information, sampling metadata, GenBank accession numbers and references of mitochondrial genomes used in the study.

Sample ID	Subfamily	Genus	Species	Sampling Metadata	Life Stage	Accession No	Reference
ZJ497	Orthocladiinae	*Rheocricotopus*	*Rheocricotopus villiculus*	Tianmu Mountain National Nature Reserve, Hangzhou, Zhejiang, China, 30.3222° N, 119.442° E, 22-July-2019, leg. X.-L. Lin	Adult male	MW373526	[33]
CNUISI-020005203	Chironominae	*Chironomus*	*Chironomus flaviplumus*	Yeondeung stream, Yeosu, South Korea 34°45′26.0″ N, E 127°42′51.2″ E, May-2020	Larva	MW770891	[42]
JN861749	Chironominae	*Chironomus*	*Chironomus tepperi*	NA	NA	JN861749	[30]
XL3993	Chironominae	*Microchironomus*	*Microchironomus tabarui*	Hengshui, Hebei, China, 37.651626° N, 115.650831° E, 1-September-2020	Adult male	MZ261913	[35]
BSZ13	Chironominae	*Polypedilum*	*Polypedilum unifascium*	Lishui, Zhejiang, China, 27°45′16″ N, 119°11′15″ E, August-2020	Larva	MW677959	[34]
KT251040	Chironominae	*Polypedilum*	*Polypedilum vanderplanki*	rock pool, Nigeria, 11.088821° N, 7.734533° E	NA	KT251040	[31]
XL690	Chironominae	*Stenochironomus*	*Stenochironomus gibbus*	Trondheim, Norway, 63.4224° N, 10.3451° E, leg. X.-L. Lin	Adult male	OL742440	Present study
ZJ761	Chironominae	*Stenochironomus*	*Stenochironomus okialbus*	Xianju, Taizhou, Zhejiang, 28.674° N, 120.600° E, November-2019, leg. X.-L. Lin	Larva	OL753645	Present study
MYK13	Chironominae	*Stenochironomus*	*Stenochironomus* sp. 1CZ	Zunyi, Guizhou, China, 27.834° N, 107.569° E, June-2020, leg. P.-P. Li	Adult male	OL753646	Present study
NLCH802	Chironominae	*Stenochironomus*	*Stenochironomus* sp. 2CZ	Ganzhou, Jiangxi, China, 24.583° N, 114.446° E, August-2020, leg. X.-L. Lin	Larva	OL742441	Present study
XL1244	Chironominae	*Stenochironomus*	*Stenochironomus* sp. 3CZ	Fogong, Nujiang, Yunnan, China, 26.5533° N, 98.9203° E, May-2018, leg. X.-L. Lin	Adult male	OL753647	Present study
XL1443	Chironominae	*Stenochironomus*	*Stenochironomus tobaduodecimus*	Ledong, Hainan, China, 18.6927° N, 108.7960° E, March-2016, leg. B.-J. Sun	Adult male	OL753648	Present study
DWS114	Chironominae	*Stenochironomus*	*Stenochironomus zhengi*	Pingbian, Honghe, Yunnan, China, 22.913178° N, 103.695553° E, leg. L.-Z. Meng	Adult male	OL753649	Present study

**Table 2:** Nucleotide composition of mitochondrial genomes of the seven *Stenochironomus* species.

	Species	Whole Genome	Protein Coding Genes	1st Codon Position	2nd Codon Position	3rd Codon Position	tRNA Genes	12S rRNA	16S rRNA	Control Region
A+T%	*Stenochironomus gibbus*	82.6	78.0	73.0	69.5	91.5	83.8	88.1	89.3	97.6
	*Stenochironomus okialbus*	82.2	77.4	73.3	69.8	89.1	84.4	87.0	89.0	96.2
	*Stenochironomus tobaduodecimus*	81.7	77.0	72.0	70.2	88.7	83.9	87.3	88.1	94.9
	*Stenochironomus zhengi*	81.8	77.8	73.0	69.8	90.7	85.1	86.1	87.2	90.6
	*Stenochironomus* sp. 1CZ	81.7	77.5	72.6	69.9	90.0	83.4	86.1	87.5	97.0
	*Stenochironomus* sp. 2CZ	82.7	79.4	74.7	70.6	93.0	84.3	88.2	89.0	98.2
	*Stenochironomus* sp. 3CZ	83.6	79.0	75.1	70.3	91.7	84.4	89.7	89.4	95.2
AT- Skew	*Stenochironomus* gibbus	0.01	−0.18	−0.05	−0.41	−0.11	0.05	−0.03	0.00	−0.08
	*Stenochironomus okialbus*	0.01	−0.19	−0.08	−0.42	−0.10	0.02	−0.09	0.01	−0.17
	*Stenochironomus tobaduodecimus*	−0.01	−0.18	−0.05	−0.41	−0.10	0.03	−0.06	−0.04	−0.09
	*Stenochironomus zhengi*	−0.02	−0.21	−0.08	−0.41	−0.15	0.02	−0.08	0.00	0.06
	*Stenochironomus* sp. 1CZ	0.01	−0.19	−0.07	−0.41	−0.10	0.03	−0.08	0.01	−0.02
	*Stenochironomus* sp. 2CZ	0.01	−0.18	−0.08	−0.41	−0.09	0.05	−0.06	0.01	−0.11
	*Stenochironomus* sp. 3CZ	0.02	−0.18	−0.08	−0.41	−0.09	0.06	−0.05	0.01	−0.09
GC- Skew	*Stenochironomus gibbus*	−0.24	0.00	0.18	−0.11	−0.18	−0.15	−0.33	−0.46	−0.33
	*Stenochironomus okialbus*	−0.22	−0.03	0.19	−0.14	−0.23	−0.13	−0.33	−0.42	0.32
	*Stenochironomus tobaduodecimus*	−0.34	−0.04	0.12	−0.13	−0.20	−0.16	−0.40	−0.43	−0.02
	*Stenochironomus zhengi*	−0.27	−0.04	0.13	−0.13	−0.23	−0.15	−0.25	−0.45	−0.55
	*Stenochironomus* sp. 1CZ	−0.29	−0.01	0.18	−0.14	−0.16	−0.16	−0.38	−0.46	−0.50
	*Stenochironomus* sp. 2CZ	−0.21	0.02	0.22	−0.13	−0.07	−0.12	−0.27	−0.45	0.00
	*Stenochironomus* sp. 3CZ	−0.24	−0.01	0.20	−0.13	−0.18	−0.14	−0.22	−0.36	0.00

## Data Availability

The following information was supplied regarding the availability of DNA sequences: The new mitogenomes of *Stenochironomus gibbus*, *Stenochironomus okialbus*, *Stenochironomus* sp. 1CZ, *Stenochironomus* sp. 2CZ, *Stenochironomus* sp. 3CZ, *Stenochironomus tobaduodecimus*, *Stenochironomus zhengi* are deposited in GenBank of NCBI under accession numbers OL742440, OL753645, OL753646, OL742441, OL753647, OL753648 and OL753649, respectively.

## Supplementary Materials

The following supporting information can be downloaded at: https://www.mdpi.com/article/10.3390/insects13020115/s1, Supplementary Table S1: The best model for each partition of the three datasets. Supplementary Table S2: Start and stop codons of PCGs among the seven *Stenochironomus* mitogenomes. Supplementary Table S3: Total number of codons of the seven *Stenochironomus* mitogenomes. Supplementary Figure S1: Patterns of codon usage of the seven *Stenochironomus* mitogenomes. The *X*-axis shows the codon families and the *Y*-axis shows the total codons. Supplementary Figure S2: Phylogenetic relationships of Chironominae based on AA. Numbers at the nodes are ML bootstrap values. Supplementary Figure S3: Phylogenetic relationships of Chironominae based on PCG12. Numbers at the nodes are BI posterior probabilities and ML bootstrap values. Supplementary Figure S4: Phylogenetic relationships of Chironominae based on PCG123. Numbers at the nodes are BI posterior probabilities and ML bootstrap values.

## References

[1] Cameron S.L. (2014). Insect mitochondrial genomics: Implications for evolution and phylogeny. Annu. Rev. Entomol..

[2] Moreno-Carmona M., Cameron S.L., Quiroga C.F.P. (2021). How are the mitochondrial genomes reorganized in Hexapoda? Differential evolution and the first report of convergences within Hexapoda. Gene.

[3] Brown W.M. (1985). The mitochondrial genome of animals. Molecular Evolutionary Genetics.

[4] Boore J.L. (1999). Animal mitochondrial genomes. Nucleic Acids Res..

[5] Brown W.M., George M., Wilson A.C. (1979). Rapid evolution of animal mitochondrial DNA. Proc. Natl. Acad. Sci. USA.

[6] Curole J.P., Kocher T.D. (1999). Mitogenomics: Digging deeper with complete mitochondrial genomes. Trends Ecol. Evol..

[7] Du Z., Hasegawa H., Cooley J.R., Simon C., Yoshimura J., Cai W., Sota T., Li H. (2019). Mitochondrial genomics reveals shared phylogeographic patterns and demographic history among three periodical cicada species groups. Mol. Biol. Evol..

[8] Kieran T.J. (2020). Mitochondrial, metagenomic, and phylogenetic analysis of the ground beetle *Harpalus pensylvanicus* (Coleoptera: Carabidae). Gene.

[9] Manchola O.F.S., Virrueta Herrera S., D’Alessio L.M., Yoshizawa K., Garcia Aldrete A.N., Johnson K.P. (2021). Mitochondrial genomes within bark lice (Insecta: Psocodea: Psocomorpha) reveal novel gene rearrangements containing phylogenetic signal. Syst. Entomol..

[10] Rokas A., Holland P.W. (2000). Rare genomic changes as a tool for phylogenetics. Trends Ecol. Evol..

[11] Zhang J., Kan X., Miao G., Hu S., Sun Q., Tian W. (2020). qMGR: A new approach for quantifying mitochondrial genome rearrangement. Mitochondrion.

[12] Zheng B.-Y., Cao L.-J., Tang P., van Achterberg K., Hoffmann A.A., Chen H.-Y., Chen X.-X., Wei S.-J. (2018). Gene arrangement and sequence of mitochondrial genomes yield insights into the phylogeny and evolution of bees and sphecid wasps (Hymenoptera: Apoidea). Mol. Phylogenet. Evol..

[13] Sweet A.D., Johnson K.P., Cao Y., de Moya R.S., Skinner R.K., Tan M., Herrera S.V., Cameron S.L. (2021). Structure, gene order, and nucleotide composition of mitochondrial genomes in parasitic lice from *Amblycera*. Gene.

[14] Dietrich C., Brune A. (2016). The complete mitogenomes of six higher termite species reconstructed from metagenomic datasets (*Cornitermes* sp., *Cubitermes ugandensis*, *Microcerotermes parvus*, *Nasutitermes corniger*, *Neocapritermes taracua*, and *Termes hospes*). Mitochondrial DNA Part. A.

[15] Zhang J., Zhou C., Gai Y., Song D., Zhou K. (2008). The complete mitochondrial genome of *Parafronurus youi* (Insecta: Ephemeroptera) and phylogenetic position of the *Ephemeroptera*. Gene.

[16] Zhang W., Li R., Zhou C. (2021). Complete mitochondrial genomes of *Epeorus carinatus* and *E. dayongensis* (Ephemeroptera: Heptageniidae): Genomic comparison and phylogenetic inference. Gene.

[17] Shi A., Li H., Bai X., Dai X., Chang J., Guilbert E., Cai W. (2012). The complete mitochondrial genome of the flat bug *Aradacanthia heissi* (Hemiptera: Aradidae). Zootaxa.

[18] Li H., Leavengood Jr J.M., Chapman E.G., Burkhardt D., Song F., Jiang P., Liu J., Zhou X., Cai W. (2017). Mitochondrial phylogenomics of Hemiptera reveals adaptive innovations driving the diversification of true bugs. Proc. R. Soc. B Biol. Sci..

[19] Cheng Y., Yan Y., Wei M., Niu G. (2021). Characterization of mitochondrial genomes of three new species: *Leptocimbex praiaformis*, *L. clavicornis*, and *L. yanniae* (Hymenoptera: Cimbicidae). Entomol. Res..

[20] Hu J., Zhang D., Hao J., Huang D., Cameron S., Zhu C. (2010). The complete mitochondrial genome of the yellow coaster, *Acraea issoria* (Lepidoptera: Nymphalidae: Heliconiinae: Acraeini): Sequence, gene organization and a unique tRNA translocation event. Mol. Biol. Rep..

[21] Ye F., Lan X.-E., Zhu W.-B., You P. (2016). Mitochondrial genomes of praying mantises (Dictyoptera, Mantodea): Rearrangement, duplication, and reassignment of tRNA genes. Sci. Rep..

[22] Zhang L.-P., Cai Y.-Y., Yu D.-N., Storey K.B., Zhang J.-Y. (2018). Gene characteristics of the complete mitochondrial genomes of *Paratoxodera polyacantha* and *Toxodera hauseri* (Mantodea: Toxoderidae). Peer J..

[23] Fenn J.D., Song H., Cameron S.L., Whiting M.F. (2008). A preliminary mitochondrial genome phylogeny of Orthoptera (Insecta) and approaches to maximizing phylogenetic signal found within mitochondrial genome data. Mol. Phylogenet. Evol..

[24] Leavitt J.R., Hiatt K.D., Whiting M.F., Song H. (2013). Searching for the optimal data partitioning strategy in mitochondrial phylogenomics: A phylogeny of Acridoidea (Insecta: Orthoptera: Caelifera) as a case study. Mol. Phylogenet. Evol..

[25] Cameron S.L., Johnson K.P., Whiting M.F. (2007). The mitochondrial genome of the screamer louse *Bothriometopus* (Phthiraptera: Ischnocera): Effects of extensive gene rearrangements on the evolution of the genome. J. Mol. Evol..

[26] Dickey A.M., Kumar V., Morgan J.K., Jara-Cavieres A., Shatters R.G., McKenzie C.L., Osborne L.S. (2015). A novel mitochondrial genome architecture in thrips (Insecta: Thysanoptera): Extreme size asymmetry among chromosomes and possible recent control region duplication. BMC Genom..

[27] Nelson L.A., Lambkin C.L., Batterham P., Wallman J.F., Dowton M., Whiting M.F., Yeates D.K., Cameron S.L. (2012). Beyond barcoding: A mitochondrial genomics approach to molecular phylogenetics and diagnostics of blowflies (Diptera: Calliphoridae). Gene.

[28] Beckenbach A.T., Joy J.B. (2009). Evolution of the mitochondrial genomes of gall midges (Diptera: Cecidomyiidae): Rearrangement and severe truncation of tRNA genes. Genome Biol. Evol..

[29] Wang Q., Huang J., Wu H. (2021). Mitogenomes provide insights into the phylogeny of *Mycetophilidae* (Diptera: Sciaroidea). Gene.

[30] Beckenbach A.T. (2012). Mitochondrial genome sequences of Nematocera (lower Diptera): Evidence of rearrangement following a complete genome duplication in a winter crane fly. Genome Biol. Evol..

[31] Deviatiiarov R., Kikawada T., Gusev O. (2017). The complete mitochondrial genome of an anhydrobiotic midge *Polypedilum vanderplanki* (Chironomidae, Diptera). Mitochondrial DNA Part. A.

[32] Lin X.L., Zhao Y.M., Yan L.P., Liu W.B., Bu W.J., Wang X.H., Zheng C.G. (2022). Mitogenomes provide new insights into the evolutionary history of *Prodiamesinae* (Diptera: Chironomidae). Zool. Scr..

[33] Zheng C.-G., Zhu X.-X., Yan L.-P., Yao Y., Bu W.-J., Wang X.-H., Lin X.-L. (2021). First complete mitogenomes of Diamesinae, Orthocladiinae, Prodiamesinae, Tanypodinae (Diptera: Chironomidae) and their implication in phylogenetics. Peer J..

[34] Lei T., Song C., Zhu X.-D., Xu B.-Y., Qi X. (2021). The complete mitochondrial genome of a non-biting midge *Polypedilum unifascium* (Tokunaga, 1938) (Diptera: Chironomidae). Mitochondrial DNA Part. B.

[35] Kong F.-Q., Zhao Y.-C., Chen J.-L., Lin X.-L. (2021). First report of the complete mitogenome of *Microchironomus tabarui* Sasa, 1987 (Diptera, Chironomidae) from Hebei Province, China. Mitochondrial DNA Part. B.

[36] Hiki K., Oka K., Nakajima N., Yamamoto H., Yamagishi T., Sugaya Y. (2021). The complete mitochondrial genome of the non-biting midge *Chironomus yoshimatsui* (Diptera: Chironomidae). Mitochondrial DNA Part. B.

[37] Jiang Y.-W., Zhao Y.-M., Lin X.-L. (2022). First report of the complete mitogenome of *Tanypus punctipennis* Meigen, 1818 (Diptera, Chironomidae) from Hebei Province, China. Mitochondrial DNA Part. B.

[38] Cranston P.S., Dillon M.E., Pinder L.C.V., Reiss F., Wiederholm T. (1989). The Adult Males of Chironominae (Diptera, Chironomidae) of the Holarctic Region—Keys and Diagnoses. Chironomidae of the Holarctic region. Keys and diagnoses. Part 3—Adult Males.

[39] Wantzen K.M., Wagner R. (2006). Detritus processing by invertebrate shredders: A neotropical–temperate comparison. J. North. Am. Benthol. Soc..

[40] Valente-Neto F., Koroiva R., Fonseca-Gessner A.A., de Oliveira Roque F. (2015). The effect of riparian deforestation on macroinvertebrates associated with submerged woody debris. Aquat. Ecol..

[41] Martins I., Castro D.M., Macedo D.R., Hughes R.M., Callisto M. (2021). Anthropogenic impacts influence the functional traits of Chironomidae (Diptera) assemblages in a neotropical savanna river basin. Aquat. Ecol..

[42] Park K., Kim W.-S., Park J.-W., Kwak I.-S. (2021). Complete mitochondrial genome of *Chironomus flaviplumus* (Diptera: Chironomidae) collected in Korea. Mitochondrial DNA Part. B.

[43] Bolger A.M., Lohse M., Usadel B. (2014). Trimmomatic: A flexible trimmer for Illumina sequence data. Bioinformatics.

[44] Peng Y., Leung H.C., Yiu S.-M., Chin F.Y. (2012). IDBA-UD: A *de novo* assembler for single-cell and metagenomic sequencing data with highly uneven depth. Bioinformatics.

[45] Kearse M., Moir R., Wilson A., Stones-Havas S., Cheung M., Sturrock S., Buxton S., Cooper A., Markowitz S., Duran C. (2012). Geneious Basic: An integrated and extendable desktop software platform for the organization and analysis of sequence data. Bioinformatics.

[46] Grant J.R., Stothard P. (2008). The CGView Server: A comparative genomics tool for circular genomes. Nucleic Acids Res..

[47] Kumar S., Stecher G., Li M., Knyaz C., Tamura K. (2018). MEGA X: Molecular evolutionary genetics analysis across computing platforms. Mol. Biol. Evol..

[48] Rozas J., Ferrer-Mata A., Sánchez-DelBarrio J.C., Guirao-Rico S., Librado P., Ramos-Onsins S.E., Sánchez-Gracia A. (2017). DnaSP 6: DNA sequence polymorphism analysis of large data sets. Mol. Biol. Evol..

[49] Vaidya G., Lohman D.J., Meier R. (2011). SequenceMatrix: Concatenation software for the fast assembly of multi-gene datasets with character set and codon information. Cladistics.

[50] Lanfear R., Frandsen P.B., Wright A.M., Senfeld T., Calcott B. (2017). PartitionFinder 2: New methods for selecting partitioned models of evolution for molecular and morphological phylogenetic analyses. Mol. Biol. Evol..

[51] Ronquist F., Teslenko M., van der Mark P., Ayres D.L., Darling A., Höhna S., Larget B., Liu L., Suchard M.A., Huelsenbeck J.P. (2012). MrBayes 3.2: Efficient Bayesian phylogenetic inference and model choice across a large model space. Syst. Biol..

[52] Rambaut A., Drummond A.J., Xie D., Baele G., Suchard M.A. (2018). Posterior summarization in *Bayesian phylogenetics* using Tracer 1.7. Syst. Biol..

[53] Nguyen L.-T., Schmidt H.A., Von Haeseler A., Minh B.Q. (2015). IQ-TREE: A fast and effective stochastic algorithm for estimating maximum-likelihood phylogenies. Mol. Biol. Evol..

[54] de Oliveira Aragão A., Neto J.P.N., Cruz A.C.R., Casseb S.M.M., Cardoso J.F., da Silva S.P., Ishikawa E.A.Y. (2019). Description and phylogeny of the mitochondrial genome of *Sabethes chloropterus*, *Sabethes glaucodaemon* and *Sabethes belisarioi* (Diptera: Culicidae). Genomics.

[55] Li X.-Y., Yan L.-P., Pape T., Gao Y.-Y., Zhang D. (2020). Evolutionary insights into bot flies (Insecta: Diptera: Oestridae) from comparative analysis of the mitochondrial genomes. Int. J. Biol. Macromol..

[56] Lorenz C., Alves J.M., Foster P.G., Suesdek L., Sallum M.A.M. (2021). Phylogeny and temporal diversification of mosquitoes (Diptera: Culicidae) with an emphasis on the Neotropical fauna. Syst. Entomol..

[57] Thao M.L., Baumann L., Baumann P. (2004). Organization of the mitochondrial genomes of whiteflies, aphids, and psyllids (Hemiptera, Sternorrhyncha). BMC Evol. Biol..

[58] Tan H.-W., Liu G.-H., Dong X., Lin R.-Q., Song H.-Q., Huang S.-Y., Yuan Z.-G., Zhao G.-H., Zhu X.-Q. (2011). The complete mitochondrial genome of the Asiatic cavity-nesting honeybee *Apis cerana* (Hymenoptera: Apidae). PLoS ONE.

[59] Jiang P., Li H., Song F., Cai Y., Wang J., Liu J., Cai W. (2016). Duplication and remolding of tRNA genes in the mitochondrial genome of *Reduvius tenebrosus* (Hemiptera: Reduviidae). Int. J. Mol. Sci..

[60] Liu Y., Li H., Song F., Zhao Y., Wilson J.J., Cai W. (2019). Higher-level phylogeny and evolutionary history of Pentatomomorpha (Hemiptera: Heteroptera) inferred from mitochondrial genome sequences. Syst. Entomol..

