# Supplementary material for: First Report on Mitochondrial Gene Rearrangement in Non-Biting Midges, Revealing a Synapomorphy in Stenochironomus Kieffer (Diptera: Chironomidae)
Source: Insects. 2022 Jan 21;13(2):115. doi: 10.3390/insects13020115 (PMC8875173; doi:10.3390/insects13020115)
Supplement: Supplementary file 1 [file insects-13-00115-s001.zip › insects-1495751-supplementary.pdf]

Supplementary materials

Table S1. The best model for each partition of the three datasets.

Datasets	Partition names	Best model
PCG123	COI, COII, COIII, CytB, ATP6, ND3	GTR+I+G
	ND2, ND6, ATP8	GTR+I+G
	ND5, ND1, ND4, ND4L	TIM+I+G
PCG12	COI, COII, ATP6, COIII, CytB	GTR+I+G
	ND2, ND3, ND6, ATP8	TVM+I+G
	ND5, ND1, ND4, ND4L	TIM+I+G
AA	COI, COII, ATP6, COIII, CytB	MTART+I+G+F
	ND2, ND3, ND6, ATP8	MTART+G+F
	ND5, ND1, ND4, ND4L	MTART+I+G+F

Table S2. Start and stop codons of PCGs among the seven *Stenochironomus* mitogenomes.

Feature	Start/stop codon						
	S. gibbus	S. *okialbus*	*S. tobaduodecimus*	*S. zhengi*	*S.* sp. 1CZ	*S.* sp. 2CZ	*S.* sp. 3CZ
ATP6	ATG\TAA	ATG\TAA	ATG\TAA	ATG\TAA	ATG\TAA	ATG\TAA	ATG\TAA
ATP8	ATT\TAA	ATT\TAA	ATA\TAA	ATT\TAA	ATT\TAA	ATT\TAA	ATT\TAA
COI	ATG\TAA	ATG\TAA	ATG\TAA	TTG\TAA	TTG\TAA	ATG\TAA	ATG\TAA
COII	ATG\TAA	ATG\TAA	ATG\TAA	ATG\TAA	ATG\TAA	ATG\TAA	ATG\TAA
COIII	ATG\TAA	ATG\TAA	ATG\TAA	ATA\TAA	ATG\TAA	ATA\TAA	ATG\TAA
CytB	ATG\TAA	ATG\TAA	ATG\TAA	ATG\TAA	ATG\TAA	ATG\TAA	ATG\TAA
ND1	ATT\TAA	ATT\TAA	ATT\TAA	ATATAA	ATT\TAA	ATG\TAA	ATT\TAA
ND2	ATT\TAA	ATT\TAA	ATT\TAA	ATT\TAA	ATT\TAA	ATT\TAA	ATT\TAA

ND3	ATA\TAA	ATT\TAA	ATC\TAA	ATT\TAA	ATT\TAA	ATT\TAA	ATT\TAA
ND4	ATG\TAA	ATG\TAA	ATG\TAA	ATG\TAA	ATG\TAA	ATG\TAA	ATG\TAG
ND4L	ATG\TAA	ATG\TAA	ATG\TAA	ATG\TAA	ATG\TAA	ATG\TAA	ATG\TAA
ND5	ATG\TAA	ATG\TAA	ATG\TAA	GTG\TAA	GTG\TAA	ATG\TAA	GTG\TAA
ND6	ATT\TAA	ATT\TAA	ATA\TAA	ATA\TAA	ATA\TAA	ATA\TAA	ATA\TAA

Table S3. Total number of codons of the seven *Stenochironomus* mitogenomes.

Species	*S. gibbus*	S. *okialbus*	*S. tobaduodecimus*	*S. zhengi*	*S.* sp. 1CZ	*S.* sp. 2CZ	*S.* sp. 3CZ
Codon numbers	3,713	3,708	3,711	3,715	3,707	3,711	3,716

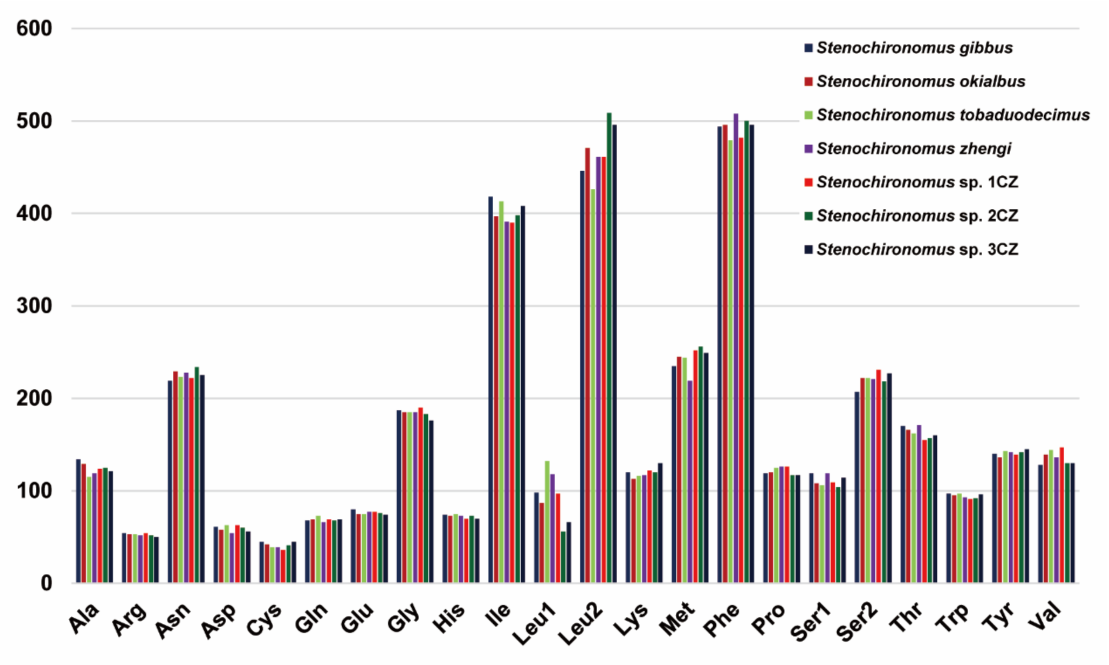

Figure S1. Patterns of codon usage of the seven *Stenochironomus* mitogenomes.

The X-axis shows the codon families and the Y-axis shows the total codons.

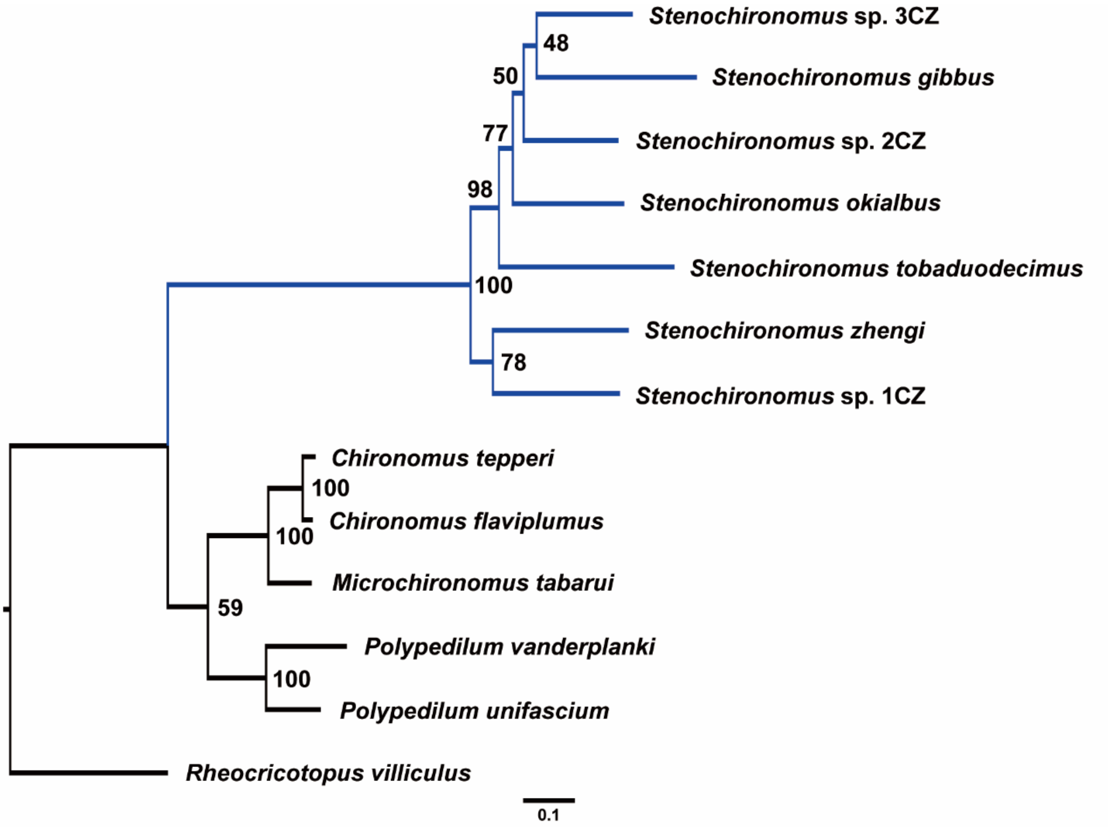

Figure S2. Phylogenetic relationships of Chironominae based on AA. Numbers at the nodes are ML bootstrap values.

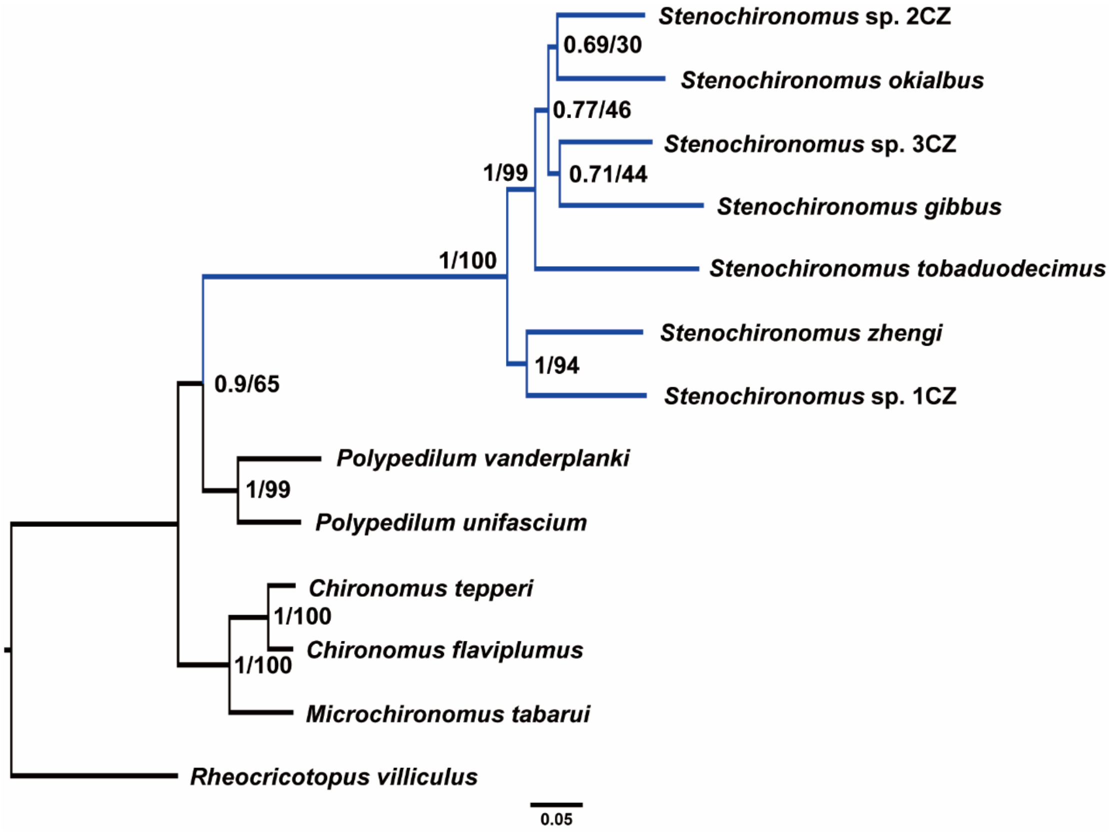

Figure S3. Phylogenetic relationships of Chironominae based on PCG12. Numbers at the nodes are BI posterior probabilities and ML bootstrap values.

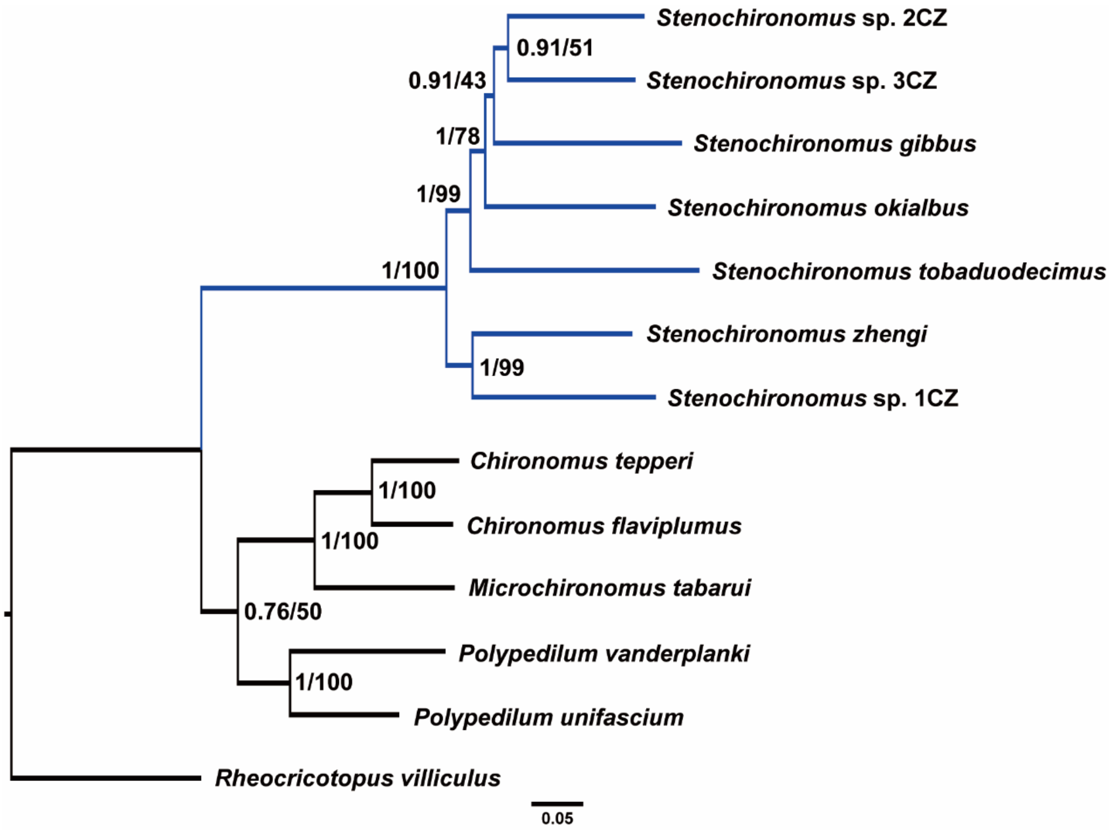

Figure S4. Phylogenetic relationships of Chironominae based on PCG123. Numbers at the nodes are BI posterior probabilities and ML bootstrap values.
